# Maternal developmental history alters transfer of circadian clock genes to offspring in Japanese quail (*Coturnix japonica*)

**DOI:** 10.1007/s00359-023-01666-2

**Published:** 2023-08-17

**Authors:** Jessica Harvey-Carroll, Tyler J. Stevenson, Karen A. Spencer

**Affiliations:** 1 School of Psychology and Neuroscience, University of St Andrews, South Street, St Andrews, KY16 9JP UK; 2 Department of Biological and Environmental Sciences, University of Gothenburg, Medicinaregatan 18A, 413 90 Gothenburg, Sweden; 3 School of Biodiversity, One Health and Veterinary Medicine, University of Glasgow, Glasgow, G36 1QH UK

**Keywords:** Circadian, Maternal transfer, Clock genes, Avian, Circadian ontogeny

## Abstract

Maternal signals shape embryonic development, and in turn post-natal phenotypes. RNA deposition is one such method of maternal signalling and circadian rhythms are one trait thought to be maternally inherited, through this mechanism. These maternal circadian gene transcripts aid development of a functioning circadian system. There is increasing evidence that maternal signals can be modified, depending on prevailing environmental conditions to optimise offspring fitness. However, currently, it is unknown if maternal circadian gene transcripts, and consequently early embryonic gene transcription, are altered by maternal developmental conditions. Here, using avian mothers who experienced either pre-natal corticosterone exposure, and/or post-natal stress as juveniles we were able to determine the effects of the timing of stress on downstream circadian RNA deposition in offspring. We demonstrated that maternal developmental history does indeed affect transfer of offspring circadian genes, but the timing of stress was important. Avian mothers who experienced stress during the first 2 weeks of post-natal life increased maternally deposited transcript levels of two core circadian clock genes, *BMAL1* and *PER2*. These differences in transcript levels were transient and disappeared at the point of embryonic genome transcription. Pre-natal maternal stress alone was found to elicit delayed changes in circadian gene expression. After activation of the embryonic genome, both *BMAL1* and *PER2* expression were significantly decreased. If both pre-natal and post-natal stress occurred, then initial maternal transcript levels of *BMAL1* were significantly increased. Taken together, these results suggest that developmental stress differentially produces persistent transgenerational effects on offspring circadian genes.

**Supplementary Information:**

The online version contains supplementary material available at 10.1007/s00359-023-01666-2.

## Introduction

Oviparous embryonic development is initially controlled exclusively by maternal gene products and proteins deposited within the egg (Abrams and Mullins 2009; Langley et al. 2014). Maternal RNA is then progressively degraded in sequential steps, whilst the zygote begins to take control of transcription (Tadros and Lipshitz 2009; Langley et al. 2014). There is increasing evidence suggesting that maternal RNA can provide active information about the maternal environment. Two recent studies using fish have shown that the critical environmental factors can also be communicated by altered maternal RNA composition in eggs. Round Goby’s (*Neogobius melanostomus*) communicate the temperature of water experienced by the mother through maternally deposited RNA. Specifically, metabolic genes such as *C1QTNF3* were found to be the main group of maternally deposited genes which displayed at least a twofold change by maternally experienced water temperature, hypothesised to optimise aspects of offspring metabolism for specific environments (Adrian-Kalchhauser et al. 2018). A further example of the environment altering maternal RNA deposition comes from Cichlid fish (from the Cichlidae family). Maternally deposited RNA of growth-related genes (for 15 haplochromine Cichlid species) were found to significantly increase or decrease depending on trophic specialisation and environment. For example, species residing in the youngest lake were found to deposit significantly lower amounts of glucocorticoid receptor transcripts within eggs. The results were hypothesised to then influence embryonic production of growth-related genes and later adaptive responses (Ahi et al. 2018). Taken together these studies indicate that environmental conditions may shape the levels of maternal signalling via RNA deposition in a wide range of physiological systems; however, to date, there has been little exploration of this phenomenon across species or physiological traits.

One system in oviparous organisms which may be inherited is the circadian system (Delaunay et al. 2000). Circadian behaviour is critical for allowing congruence between internal and external cycling environments, facilitating optimal resource usage and energy expenditure (Mazzoccoli et al. 2012; Patke et al. 2020). Underlying circadian behaviour are molecular oscillations in clock genes and proteins that interact with each other in transcriptional–translational feedback loops. At the simplified core of these loops are *Bmal* (Brain and Muscle Arnt-like proteins) and *Per* family genes (Period). *Bmal*-related genes activate transcription of *Per* family genes, which then in turn repress *Bmal* transcription. This autoinhibitory feedback loop results in the circadian system featuring cyclic expression throughout a 24-h period. The rhythmic expression and repression of these circadian clock genes modulates downstream expression of genes associated with behavioural and physiological changes. This ‘clock system’ is ubiquitous throughout an organism and across species (Reppert and Weaver 2003; Ko and Takahashi 2006).

Circadian system ontology is thought to originate from maternally deposited RNA, specifically *Bmal* and *Per* family transcripts (Delaunay et al. 2000; Shang and Zhdanova 2007). This appears to be a conserved developmental mechanism. Maternal transcripts of circadian genes have been identified in oocytes and early embryonic stages prior to activation of the zygotic genome in mammals (*Mus musculus, Oryctolagus cuniculus*), fish (*Perca fluviatilis* and *Danio rerio*), birds (*Gallus gallus*) and amphibians (*Xenopus laevis)* (Delaunay et al. 2000; Curran et al. 2008; Dekens and Whitmore 2008; Amano et al. 2009, 2010; Gonçalves et al. 2012; Nishikawa et al. 2013; Almeida et al. 2019). It has been suggested that maternally deposited circadian genes may be involved in phase synchronisation of zygotic clock gene transcription. For example, accumulation of maternal *Per3* was suggested to aid initiation of the phase of the free running clock in zebrafish embryos (Delaunay et al. 2000).

The circadian system is sensitive to alteration by external and internal cues, allowing for adaptability. One such factor that the system is particularly sensitive to is stress (Razzoli et al. 2014; Tahara et al. 2015; Koch et al. 2017). Preliminary studies suggest pre-natal stress interacts with elements of the post-natal circadian system, such as the endogenous corticosterone rhythm (Koehl et al. 1997, 1999; Kiryanova et al. 2017). Following maternal stress, offspring have been found to feature hyperactivity, and diminished shifting abilities of circadian rhythms (Kiryanova et al. 2017; Morley-Fletcher et al. 2019). Attenuated expression of circadian clock genes and phase shifted circadian rhythms have also been identified (Yun et al. 2020). This suggests that circadian clock genes and consequently circadian behaviour is susceptible to alteration following exposure to both pre-natal and post-natal developmental stress.

As the circadian system appears to be inherited through maternal RNA, there is a strong theoretical basis for the idea that stressful maternal environments may alter circadian RNA deposition and potentially disrupt zygotic transcription of clock genes. However, this has yet to be tested.

In this study we investigated the effects of maternal developmental history on the deposition of maternal RNA and initiation of embryonic circadian gene transcription. We used a well-established comparative model of circadian neurobiology, the Japanese quail (*Coturnix japonica*) and investigated the expression of two core circadian clock genes, *Bmal1* (a main positive clock element) and *Per2* (a main negative clock element) from the point of maternal RNA deposition to early embryonic genome activation.

## Materials and methods

All procedures and housing of animals complied with the local ethics committee at the University of St. Andrews and in accordance with the Animals (Scientific Procedures) Act 1986 ASPA regulations under PIL IE1CF3B75 held by JHC and PPL 70/8159 and PAF9F705D held by KAS.

### Generation of maternal developmental environments

#### Pre-natal maternal manipulations

**Fig. 1:**
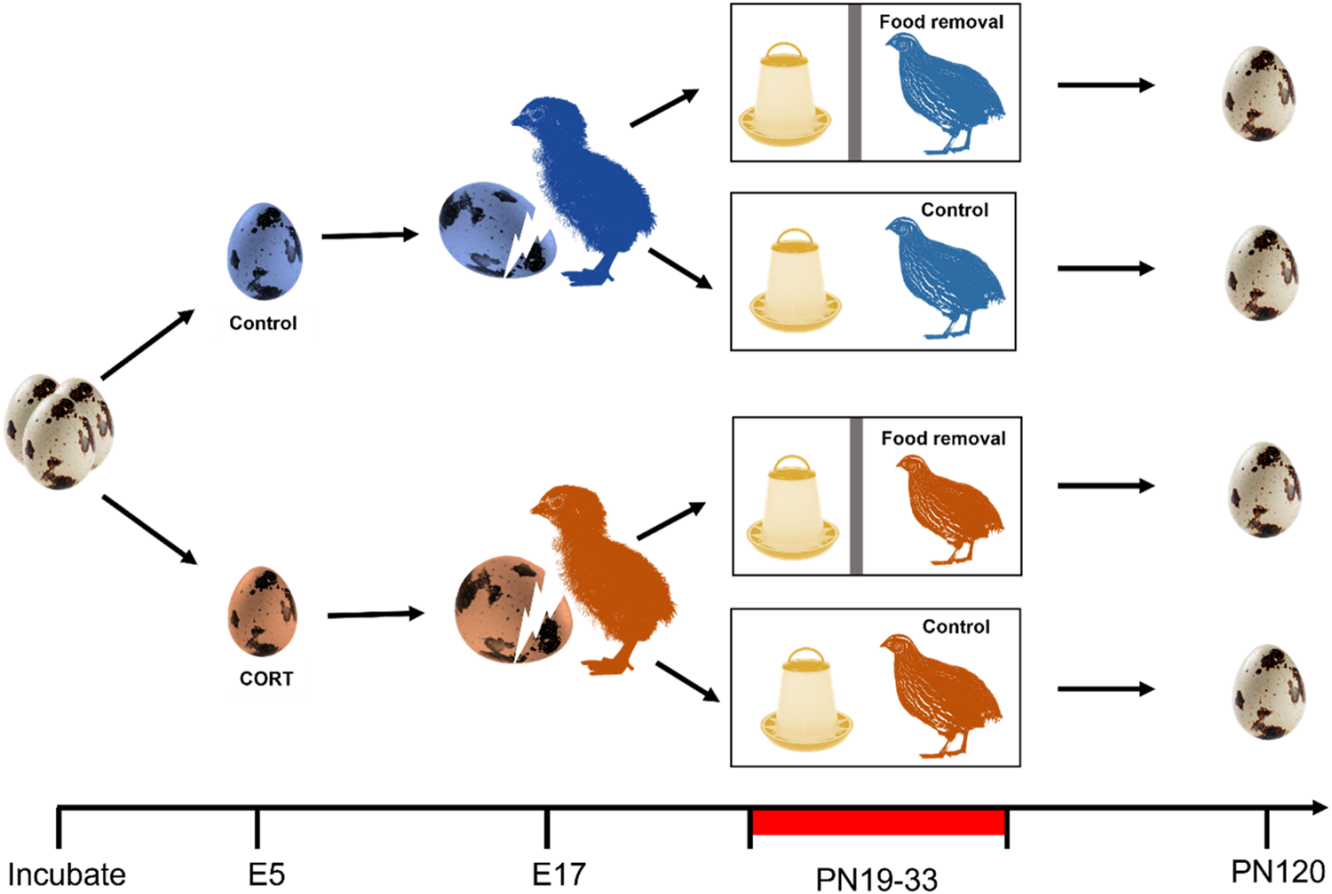
Schematic for experiment. Eggs were injected with 10 µl of 850 ng/ml CORT (orange) or 10 µL peanut oil (blue) at embryonic (E) day 5. Chicks were then subjected to an unpredictable food removal paradigm, or allowed ad libitum food access at post-natal day 19, for 14 days. The feeder and surrounding area were blocked by a panel to prevent access to food for 25% of daylight hours. Eggs collection commenced at post-natal day (PN) 120

53 fertile Japanese quail eggs (supplier Moonridge farm, Exeter, UK) were incubated (Ova-Easy 190A, Brinsea Products Ltd, UK) in complete darkness whilst on a rotating platform at 37.4 °C with 60% humidity. Fertility was confirmed at day 5 of incubation via an egg candling torch. Eggs were injected at the apex under sterile conditions, as per Zimmer et al. (2013). Injections occurred at day 5 of incubation (Fig. 1). The experimental treatment (*N* = 29) consisted of 10 µl of 850 ng/ml corticosterone (CORT) prepared in sterile peanut oil (Sigma Aldrich, Poole, UK). The total dose administered was 8.5 ng, as per Zimmer et al. 2013. Administration of 8.5 ng increases the CORT levels 1.8 SD above control CORT yolk levels (Zimmer et al. 2013).

Control eggs (*N* = 24) were injected with 10 µL sterile peanut oil. Needle punctures were sealed (Germolene New Skin, UK), eggs were labelled with their treatment and returned to incubator within 30 min of being removed. At day 13 of incubation, eggs were removed from the Ova-Easy incubator and placed in a treatment specific hatcher (Janoel24 incubator, 80% humidity) and egg turning was ceased. Hatchers were covered with black plastic to ensure the eggs were not exposed to light during the final days of incubation.

#### Quail housing

All chicks hatched within 48 h. Hatched chicks (Control *n* = 18, hatch rate = 75%; CORT *n* = 18, hatch rate = 62%) remained in the hatcher until feathers were dry, a period of approximately 24 h. After this they were transferred to 1m^2^ floor area pens. CORT and control chicks were moved at the same time into four pre-natal treatment specific pens (two pre-natal CORT and two pre-natal control with a 1 m^2^ floor area). Chicks were provided with ad libitum food (ground up chick crumb, Dodson & Horrell Ltd, UK) water and an electric contact brooder (Comfort chicks and hatchers, UK). Chicks were kept on a 12:12 light cycle at all times.

At 3 days of age, chicks were moved into larger treatment specific pens (120 cm × 180 cm). One pen from each pre-natal condition was allocated to one of two post-natal treatments; food removal or control (Fig. 1). This gave four groups with 9 chicks in each (pre-natal control/post-natal control *n* = 9, pre-natal control/post-natal stress *n* = 9, pre-natal CORT injected/post-natal control *n* = 9, pre-natal CORT injected/post-natal stress *n* = 9). In the larger pens, two electric contact brooders were provided for each pen, alongside ad libitum food (chick crumb, Dodson & Horrell Ltd, UK) and water.

#### Post-natal maternal manipulations

At 19 days of age, chicks in the post-natal stress conditions (pre-natal control *n* = 9, pre-natal CORT *n* = 9) were subjected to an unpredictable random food removal paradigm. For 25% of daylight hours food was removed unpredictably for 14 consecutive days, as per Zimmer et al. (2013). Feeders were kept in the same location and a panel was placed in front of the food to prevent access. It was ensured any scattered grain was also blocked by the panel (Fig. 1).

The experiment aimed to investigate maternal transfer of RNA. Therefore, only females were kept in each treatment once sex could be determined (pre-natal control/post-natal control: males *n* = 5; females *n* = 4. Pre-natal control/post-natal stress: males *n* = 6; females *n* = 3. Pre-natal CORT injected/post-natal control: males *n* = 5; females *n* = 4. Pre-natal CORT injected/post-natal stress: males *n* = 4; females *n* = 5.) In addition 4 control males (who had received control peanut oil injections and no post-natal food manipulation) were retained to produce fertile eggs for the generation of embryos to study maternal transfer. Of the four remaining control males, one male was placed in each treatment. Males were rotated between conditions approximately every 30 days throughout the experiment to encourage breeding interests.

#### Generation of embryos to study maternal transfer

Egg collection began at 120 days, when eggs were laid regularly for all conditions. Eggs older than 1 week were not incubated. Eggs were incubated every 6 days; the oldest eggs were 6 days, whilst the youngest were 1 day. Eggs had to be incubated in this manner to allow for sufficient sample size. Not all hens produced equally fertile eggs; therefore, collection over multiple days enabled fertile replicates from each quail to be obtained. Eggs were incubated (Ova-Easy 190A, Brinsea Products Ltd, UK) in complete darkness at 37.4 °C with 60% humidity. Eggs were not rotated whilst incubated to help stabilise the embryo for dissections.

#### Embryo dissections

The Hamburger and Hamilton (HH) staging system for chickens was used for dissections (Hamburger and Hamilton 1951; Bellairs and Osmond 2005; Hill 2021). Quail and chicken follow the same development time until HH19 (approximately 72 h) Ainsworth et al. (2010). Eggs collected from the quail were incubated for 2 h, 18 h, 23 h and 55 h, corresponding to HH stages 1, 4, 6 and 15, respectively. Maternally deposited transcripts are present at HH1. HH4 marks the onset of gastrulation, with increasing embryonic RNA (and decreasing maternal transcripts) by HH6. HH15 consists of only embryonic RNA (Gonçalves et al. 2012). Single timepoints at each developmental stage were used as clock genes do not oscillate until late embryonic development (Okabayashi et al. 2003; Csernus et al. 2007; Zeman et al. 2009).

#### Hamburger and Hamilton (HH) stages 1,4 and 6 dissections

Eggs were dissected using a stereoscopic microscope (SMZ745T, Nikon) under red light only. All dissections were carried out within the same hour. To control for order effects, dissection of control and CORT embryos were carried out alternately. Eggs were held at 5 °C prior to dissection. Blackout curtain lining (Black Blackout Thermal 3 Pass Curtain Lining Fabric, Amazon) was sealed around the microscope to ensure no light entered the dissecting area. Thin red Acetate (A4 Red Acetate 200 Micron × 5 Sheets—UK Card Crafts) was sealed around the lamp (Photonic PL2000). Red light was used as it has been demonstrated that light sensitive circadian clock genes, such as *PER2,* are least effected by red light exposure compared to other wavelengths (Di Rosa et al. 2015). A ~ 2 cm window was cut into the egg and approximately 4 ml of albumen was removed. If the embryo was not found by inspection of the yolk, dye solution (3:10 Indian ink: PBS (Lee et al. 2005) was injected under the chorion to reveal the location of the embryo to ensure precise dissection. The embryo was then dissected out of the chorion and placed immediately into RNAlater (AM7020, Invitrogen). For early developmental timepoints dissecting proto-nervous system tissue was not possible via light microscope due to accuracy. In addition, embryos prior to HH15 were not solid enough to remain intact for dissections. Therefore, the whole embryo was used. Whole embryos have been used successfully for clock gene expression in early chicken development (Gonçalves et al. 2012).

#### Hamburger and Hamilton stage 15 dissections

**Fig. 2:**
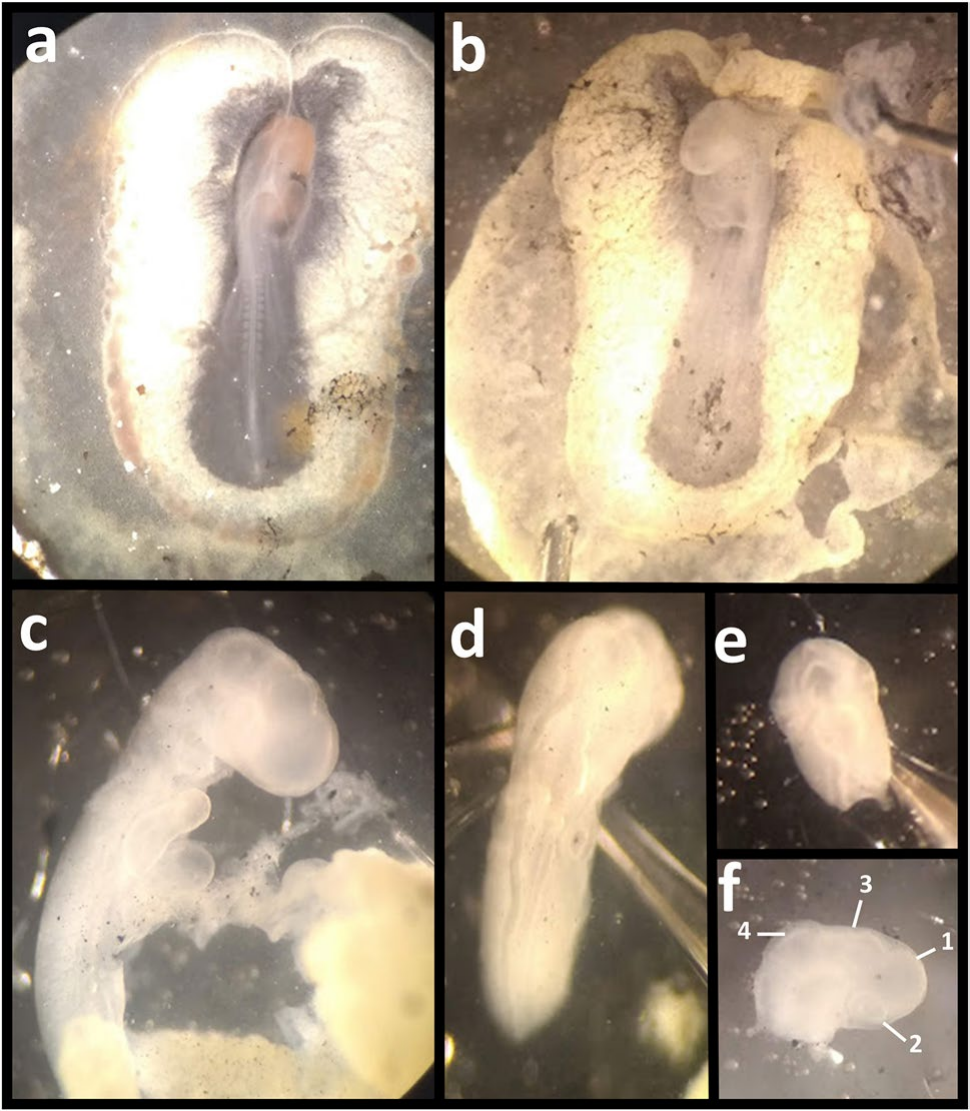
HH15 embryo dissection. **A** Egg injected with dye solution to reveal embryo. **B** Embryo dissected out and placed in dish with PBS. **C** Excess tissue removed. Embryo removed from Amnion. **D** Dorsal view of dissected embryo (posterior section discarded). **E** Dorsal view of final dissected region. **F** Side view of final dissected region. Section consists of; (1) prosencephalon, (2) optic vesicle, (3) metencephalon and (4) mesencephalon. Tissue ends just below metencephalon. Embryo was exposed to white light specifically for images

For HH15 dissections, Indian ink:PBS dye solution was injected into the egg, and the whole embryo was removed from the yolk as above. The anterior region of the embryo was dissected out to reveal the anterior portion of the embryo (Fig. 2). The anterior region of the embryo (Fig. 2F) was then removed using pulled glass capillaries (Standard Glass Capillaries—4 in, OD 1.5 mm, Filament, World Precision Instruments and Narishige PC-100). Dissected tissue was immediately placed in RNAlater.

#### RNA extraction and qPCR

Total RNA was extracted via Absolutely RNA miniprep kit (Agilent, UK) as per the manufacturer’s instructions. RNA concentrations for all samples were analysed on a QuBit 2.0 flourometer using RNA HS Assay Kit (Thermofisher, UK). A subset of 30 samples were analysed for integrity using a 2100 BioAnalyzer system, RNA 6000 nanokit (Agilent, UK). The mean RIN was 3.9 and ranged from 1 to 10.

2 ng of cDNA was then synthesised (nanoscript2 reverse transcription kits, primer design, UK). qPCR analysis was then performed using the synthesised cDNA. Specific primers were designed and validated (PrimerDesign Ltd, UK), amplifying single products only for *Bmal1* and *Per2*, *Bmal1*-Foward: GTACGTTTCTCGACATGCAATAGA Reverse: AGGTGTCCTATATCATCTTGATGGAA, *Per2*-Forward: CTGGCAAACCTGAAAGTGTTGTA Reverse: CTCCACTTGGACCATCTTCTATCA.

βactin (*ACTβ*) was used as the reference gene due to being previously identified as the most stable via gnorm (Zimmer and Spencer 2014). All qPCR runs were conducted in duplicate, with a 20 ul total reaction volume (10 uL PrecisionPLUS qPCR Master Mix, 1 uL primer mix (300 ng/20 uL reaction), 8 uL RNAse free H2O and 1uL (0.45 nG) DNA. The following amplification settings were used as per manufactures instructions (Presicion plus mastermix, primerdesign); an initial 2 min at 95 °C, and then 40 cycles of 10 s at 95 °C, 60 s at 60 °C, followed by a melt curve analysis. All standard curve efficiencies were above 96%, and *r*^2^ > 0.7. ΔCT was calculated as $\mathrm{\Delta CT}={2}^{-( \overline{x}\mathrm{ Gene of interest }- \overline{x}\mathrm{ housekeeping })}$

#### Statistical analysis

All statistics were conducted in RStudio version 1.4.17. Residuals did not follow a normal distribution, so transcript expression data sets were log transformed to fulfilled parametric model assumptions. Stability of the housekeeping gene was assessed. *ACTβ* was found to be differentially expressed between age groups: *F*_(3,127)__=_ 11.72, *P* < 0.0001. Expression was stable across treatments (Prenatal stress: *F*_(1,127)__=_ 0.510, *P* = 0.48, Postnatal stress: *F*_(1,127)_ = 0.23, *P* = 0.63). Due to unstable expression of *ACTβ* across developmental time, age specific models were conducted. To compare relative transcript expression, age specific ANOVAs were conducted for each gene. Pre-natal and post-natal treatments were included as main effects. Interactions were also included between pre-natal and post-natal treatments (gene ~ pre-natal + post-natal + pre-natal*post-natal). The room quail were housed in was included as a blocking factor in the ANOVAs (Mangiafico 2016). If significant effects were found, Tukey multiple comparisons of means with a 95% confidence was run as a post hoc test, where appropriate.

## Results

### Maternal RNA transcript deposition at HH1 is affected by developmental history

**Fig. 3:**
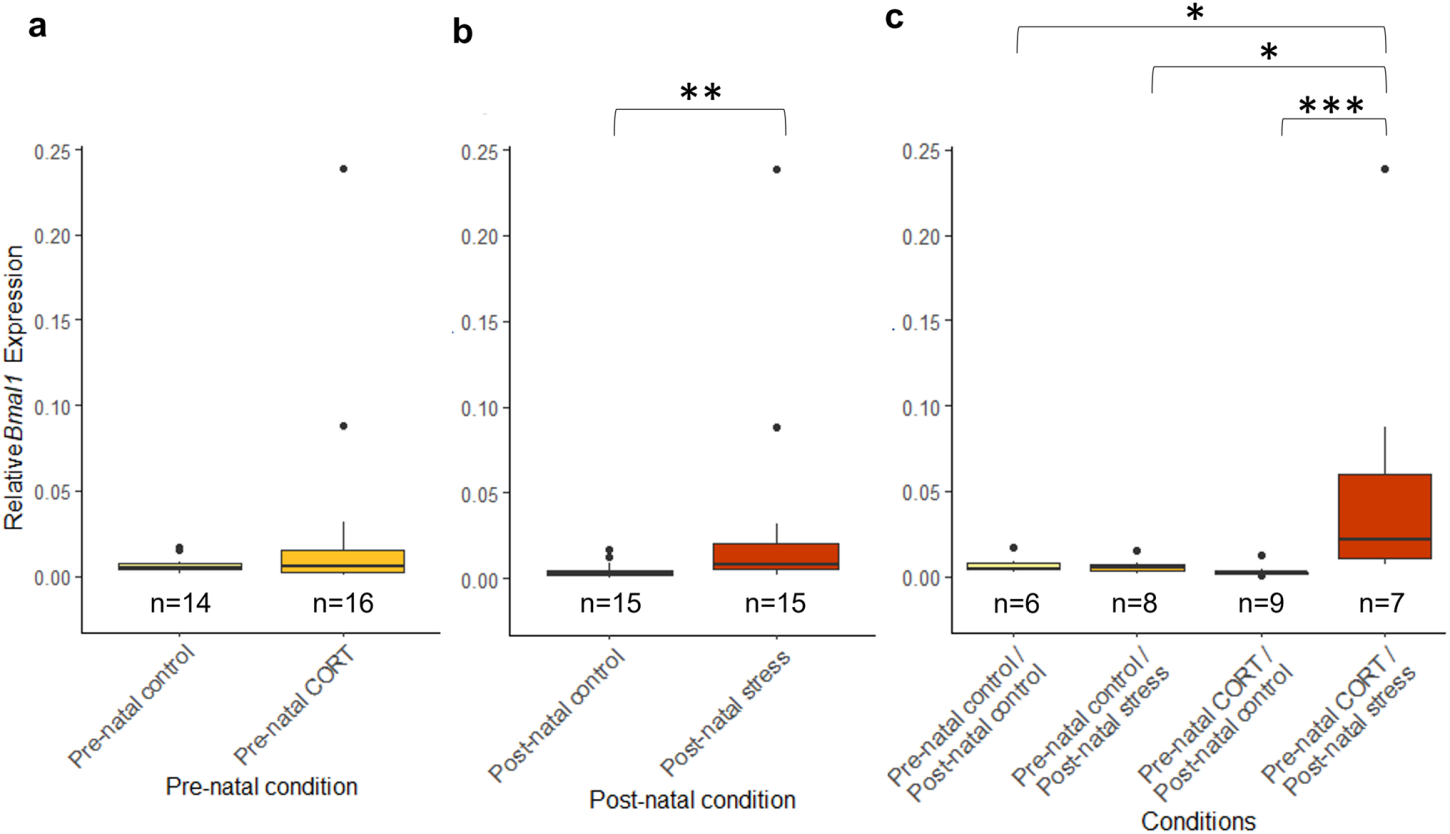
Effects of maternal developmental history on *Bmal1* gene deposition at HH1 using whole embryos. Normalized, untransformed Delta CT values are shown. **a** Pre-natal CORT exposure only. **b** Post-natal stress only. **c** Interactions between pre-natal CORT exposure and post-natal stress. Boxplots depict median relative expression levels and the 25th and 75th percentiles. Whiskers are 1.5× the interquartile range, data points outside this range are marked as outliers (circles). ***Indicate significant difference (*P* < 0.001), **Indicate significant differences (*P* < 0.01) and *Indicates significant differences (*P* < 0.05)

*Bmal1* and *Per*2 were found in all samples at HH1, 2 h after incubation. At HH1, maternal developmental history significantly affected deposition of both *Bmal1* and *Per2* in the developing embryo (Fig. 3). For *Bmal1,* a significant interaction between maternal pre-natal and post-natal stress was identified (*F*_(1,26)_ = 14.33, *P* = 0.00082). Post hoc analysis revealed a significantly higher level of *BMAL1* in embryos whose mothers had experienced both pre-natal CORT and post-natal stress, compared to all other conditions (pre-natal CORT/post-natal stress—pre-natal control/post-natal control (*P* = 0.025), pre-natal CORT/post-natal stress—pre-natal control/post-natal stress (*P* = 0.0047), and pre-natal CORT/post-natal stress—pre-natal CORT/post-natal control (*P* = 0.00015), supplementary Table 1). Pre-natal maternal stress was not found to effect *BMAL1* expression (*F*_(1,26)_ = 1.18, *P* = 0.29). Post-natal maternal stress, however, was found to significantly increase *Bmal1* expression within embryos from post-natally stressed mothers, compared to controls (*F*_(1,26)_ = 11.63, *P* = 0.0021). This marked difference is likely due to the strong increase of *Bmal1* seen in pre-natal CORT/post-natal stress embryos.

**Fig. 4:**
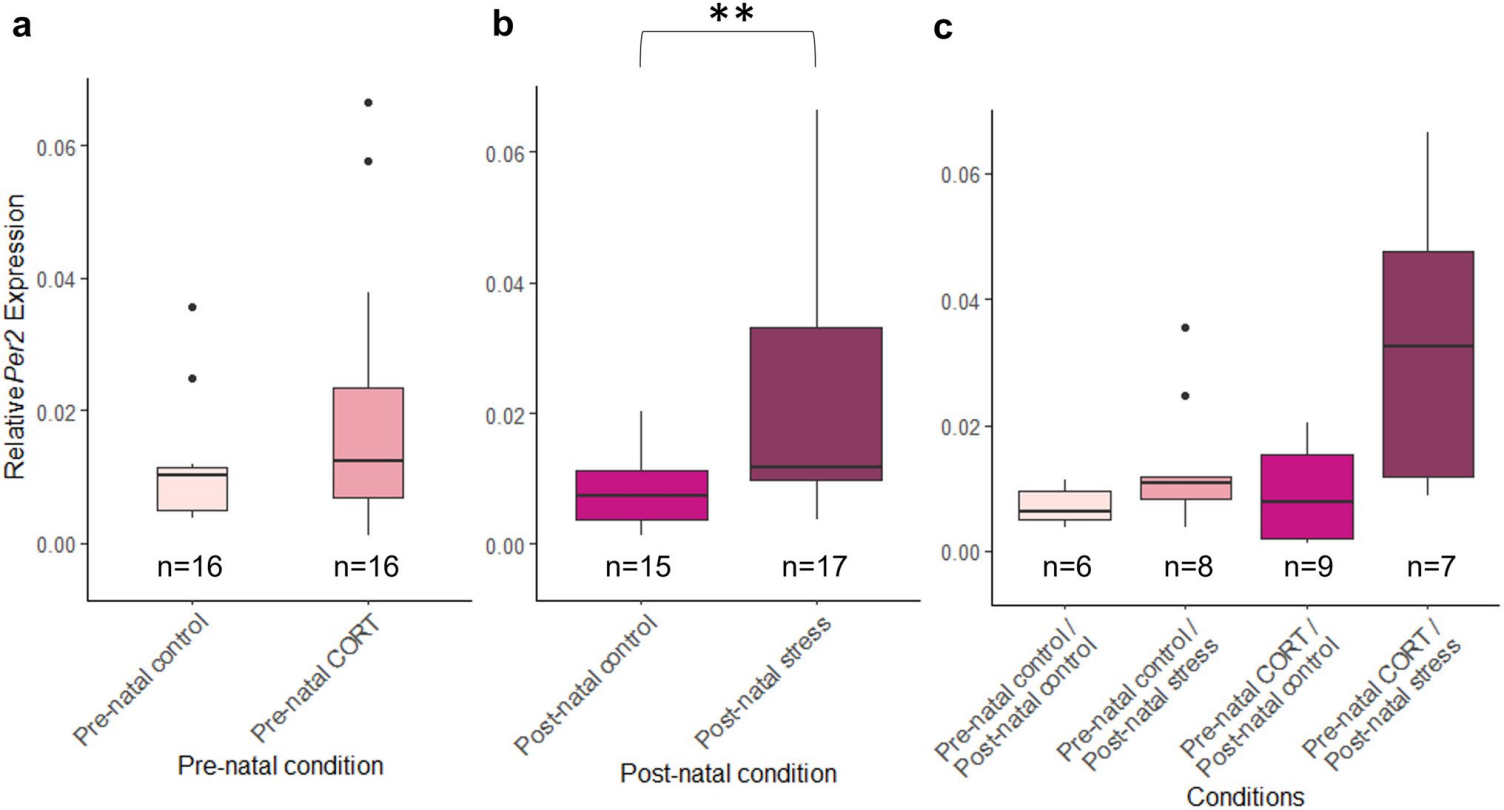
Effects of maternal developmental history on *Per2* gene deposition *at HH1* using whole embryos. Normalized, untransformed Delta CT values are shown. **a** Pre-natal CORT exposure only. **b** Post-natal stress only. **c** Interactions between pre-natal CORT exposure and post-natal stress. Boxplots depict median relative expression levels and the 25th and 75th percentiles. Whiskers are 1.5× the interquartile range, data points outside this range are marked as outliers (circles). ***Indicate significant difference (*P* < 0.001), **Indicate significant differences (*P* < 0.01) and *Indicates significant differences (*P* < 0.05)

For *Per2* expression, no significant interactions between pre-natal and post-natal stress were identified (*F*_(1,28)_ = 2.36, *P* = 0.14). Similar to *Bmal1, Per2* expression at HH1 was not significantly affected by pre-natal maternal stress (*F*_(1,28)_ = 0.3, *P* = 0.59). A significant increase in relative *Per2* expression was identified in embryos whose mothers had experienced post-natal stress (*F*_(1,28)_ = 10.15, *P* = 0.0035) (Fig. 4).

### Circadian clock gene RNA at embryonic stage HH4 is affected by maternal developmental history

**Fig. 5:**
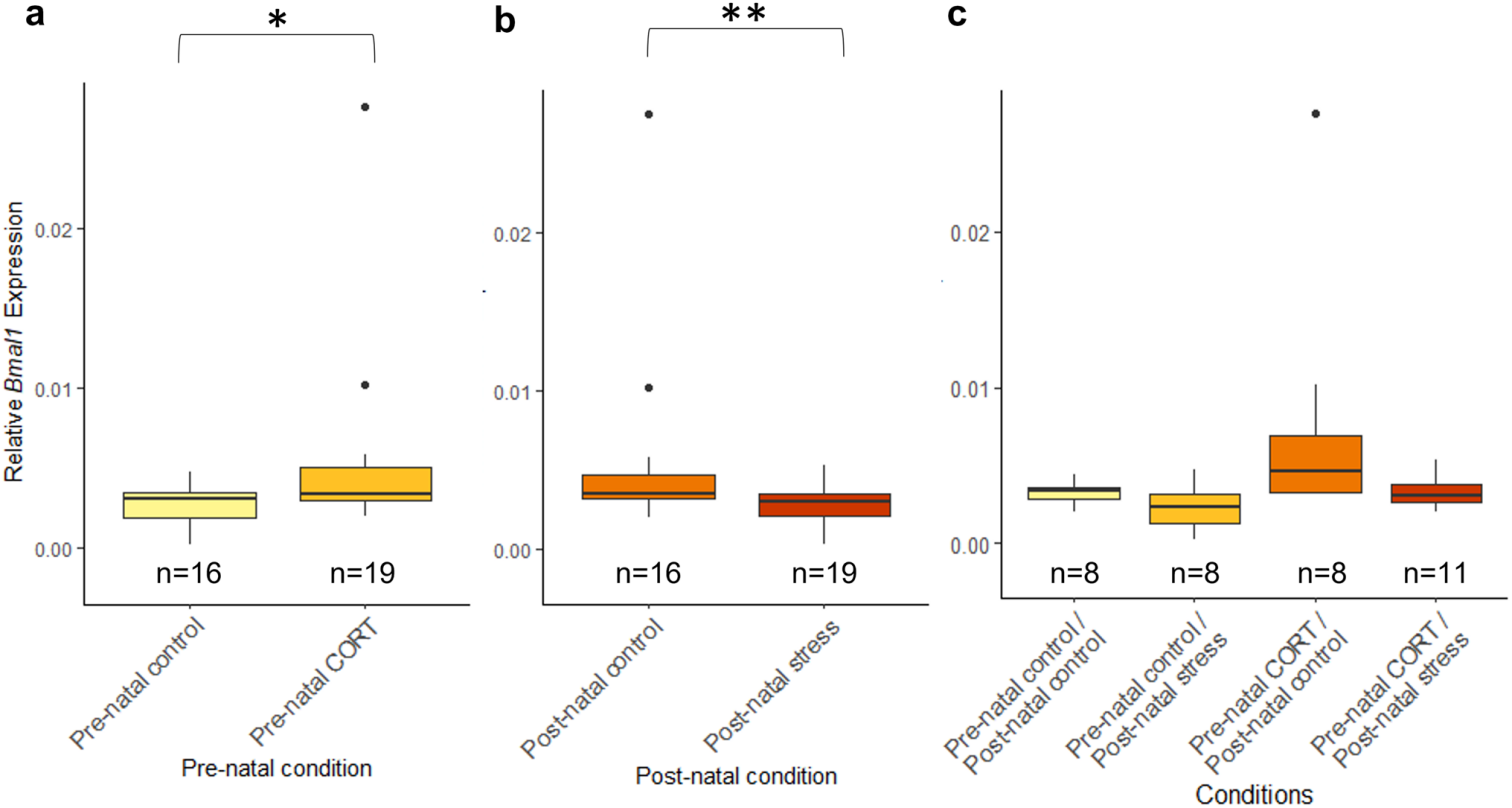
Effects of maternal developmental history on *Bmal1* relative expression at HH4 using whole embryos. Normalized, untransformed Delta CT values are shown. **a** Pre-natal stress only. **b** Post-natal stress only. **c** Interactions between pre-natal CORT exposure and post-natal stress. Boxplots depict median relative expression levels and the 25th and 75th percentiles. Whiskers are 1.5× the interquartile range, data points outside this range are marked as outliers (circles). ***Indicate significant difference (*P* < 0.001), **Indicate significant differences (*P* < 0.01) and *Indicates significant differences (*P* < 0.05)

At HH4, no significant interaction was identified between pre- and post-natal stress and *Bmal1* expression (*F*_(1,31)_ = 0.156, *P* = 0.7). *Bmal1* expression was significantly affected by the developmental timing a stress was received (Fig. 5) (pre-natal: *F*_(1,31)_ = 8.26, *P* = 0.0073, post-natal: *F*_(1,31)_ = 9.03, *P* = 0.0052). Embryos from mothers that had experienced pre-natal stress alone were found to have a significant increase in *Bmal1* expression, whereas a decreased *Bmal1* expression was found in embryos whose mothers experienced post-natal stress.

**Fig. 6:**
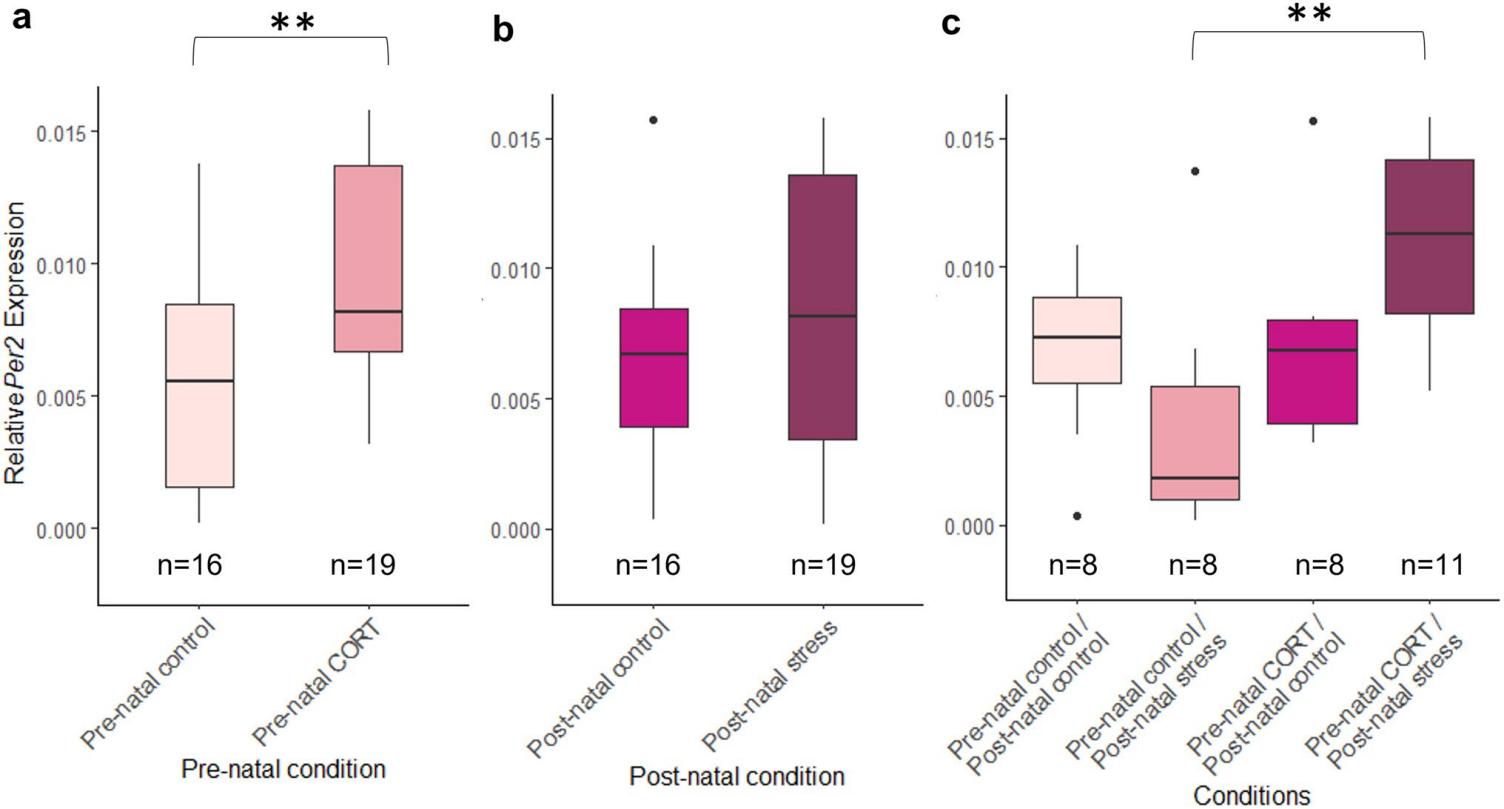
Effects of maternal developmental history on *Per2* relative expression at HH4 using whole embryos. Normalized, untransformed Delta CT values are shown. **a** Pre-natal CORT only. **b** Post-natal stress only. **c** Pre-natal and post-natal interactions. Boxplots depict median relative expression levels and the 25th and 75th percentiles. Whiskers are 1.5× the interquartile range, data points outside this range are marked as outliers. ***Indicate significant difference (*P* < 0.001), **Indicate significant differences (*P* < 0.01) and *Indicates significant differences (*P* < 0.05)

For *Per2* expression, a significant interaction between pre-natal and post-natal maternal stress was identified (*F*_(1,31)_ = 5.71, *P* = 0.023, Fig. 6). Post hoc analysis revealed *Per2* was also significantly higher in embryos from mothers who has experienced both pre-natal CORT and post-natal stress compared to those who had experienced post-natal stress alone (*P* = 0.0017) (Supplementary Table 1). Similar to *Bmal1*, *Per2* expression was significantly increased in whole embryos from pre-natal stress in mothers (*F*_(1,31)_ = 10.77, *P* = 0.0026). Post-natal stress was not found to cause any significant differences in *Per2* expression (*F*_(1,31)_ = 0.23, *P* = 0.64).

### Maternal developmental history does not alter circadian clock gene levels at HH6

**Fig. 7:**
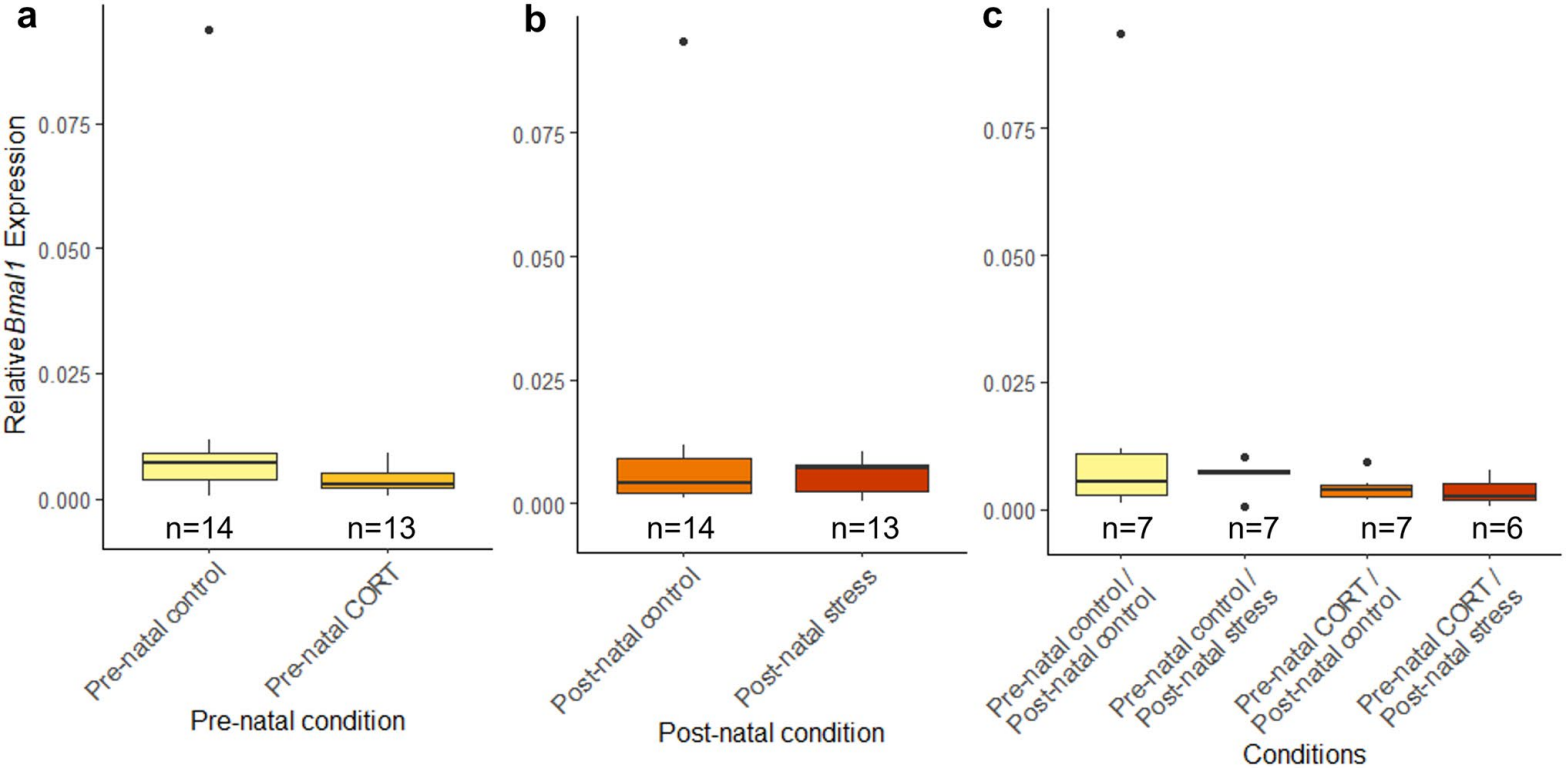
Effects of maternal developmental history on *Bmal1* gene deposition *at* HH6 using whole embryos. Normalized, untransformed Delta CT values are shown. **a** Pre-natal CORT exposure only. **b** Post-natal stress only. **c** Interactions between pre-natal CORT exposure and post-natal stress. Boxplots depict median relative expression levels and the 25th and 75th percentiles. Whiskers are 1.5X the interquartile range, data points outside this range are marked as outliers (circles). ***Indicate significant difference (*P* < 0.001), **Indicate significant differences (*P* < 0.01) and *Indicates significant differences (*P* < 0.05)

**Fig. 8:**
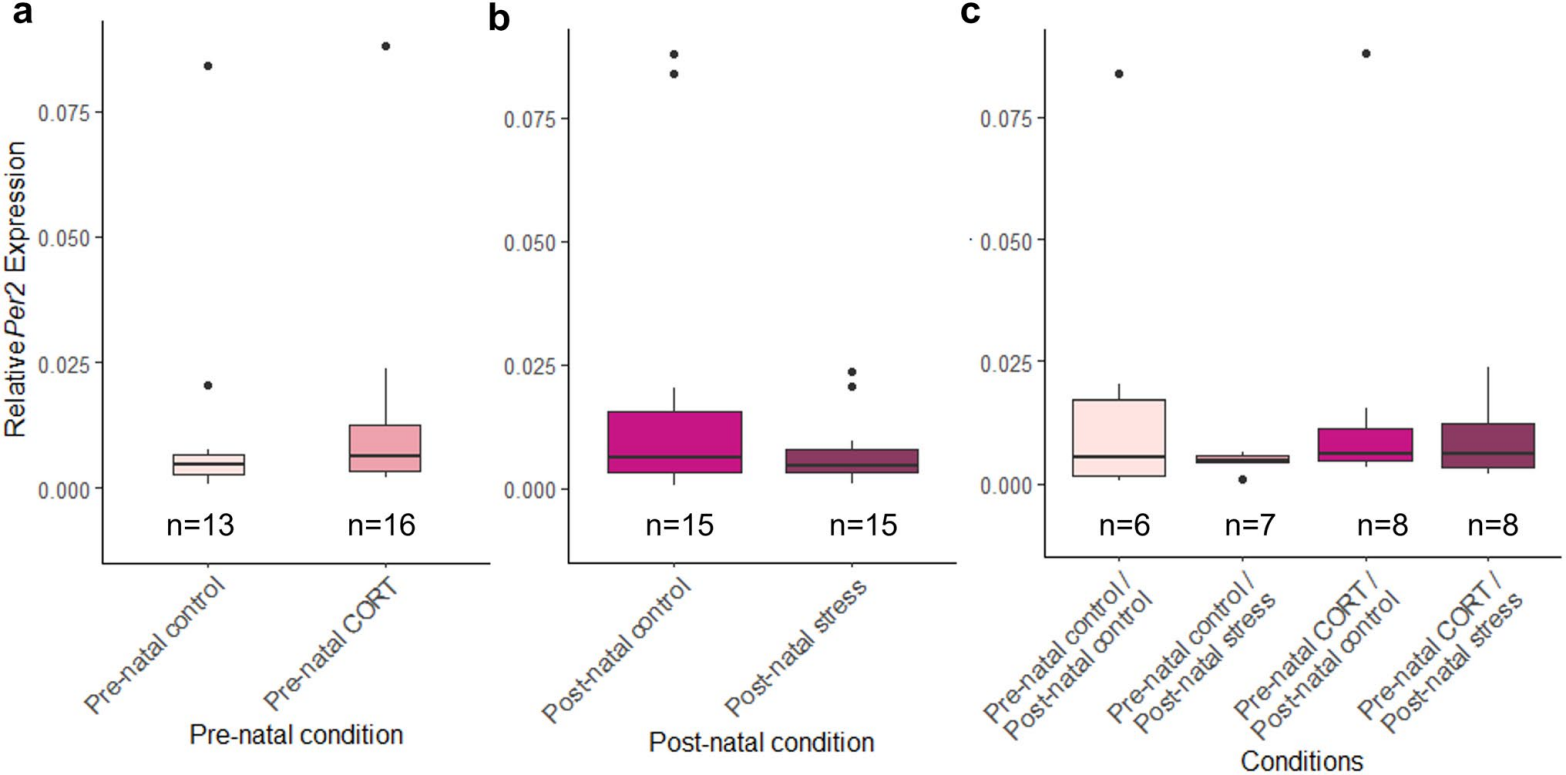
Effects of maternal developmental history on *Per2* gene deposition *at HH6* using whole embryos. Normalized, untransformed Delta CT values are shown. **a** Pre-natal CORT exposure only. **b** Post-natal stress only. **c** Interactions between pre-natal CORT exposure and post-natal stress. Boxplots depict median relative expression levels and the 25th and 75th percentiles. Whiskers are 1.5X the interquartile range, data points outside this range are marked as outliers (circles). ***Indicate significant difference (*P* < 0.001), **Indicate significant differences (*P* < 0.01) and *Indicates significant differences (*P* < 0.05)

At HH6, no significant interactions between pre-natal and post-natal stress were identified (*Bmal1*: *F*_(1,23)_ = 0.59, *P* = 0.52; Fig. 7, *Per2*: *F*_(1,25)_ = 2.07, *P* = 0.16; Fig. 8). No differences in *Bmal1* or *Per2* expression were identified at HH6 following pre-natal maternal stress (*Bmal1*: *F*_(1,23)_ = 0.53, *P* = 0.48, *Per2*: *F*_(1,25)_ = 0.2, *P* = 0.66) or post-natal maternal stress (*Bmal1*: *F*_(1,23)_ = 1.35, *P* = 0.26, *Per2*: *F*_(1,25)_ = 1.49, *P* = 0.23).

### Maternal developmental history alters embryonic circadian gene transcription at HH15

**Fig. 9:**
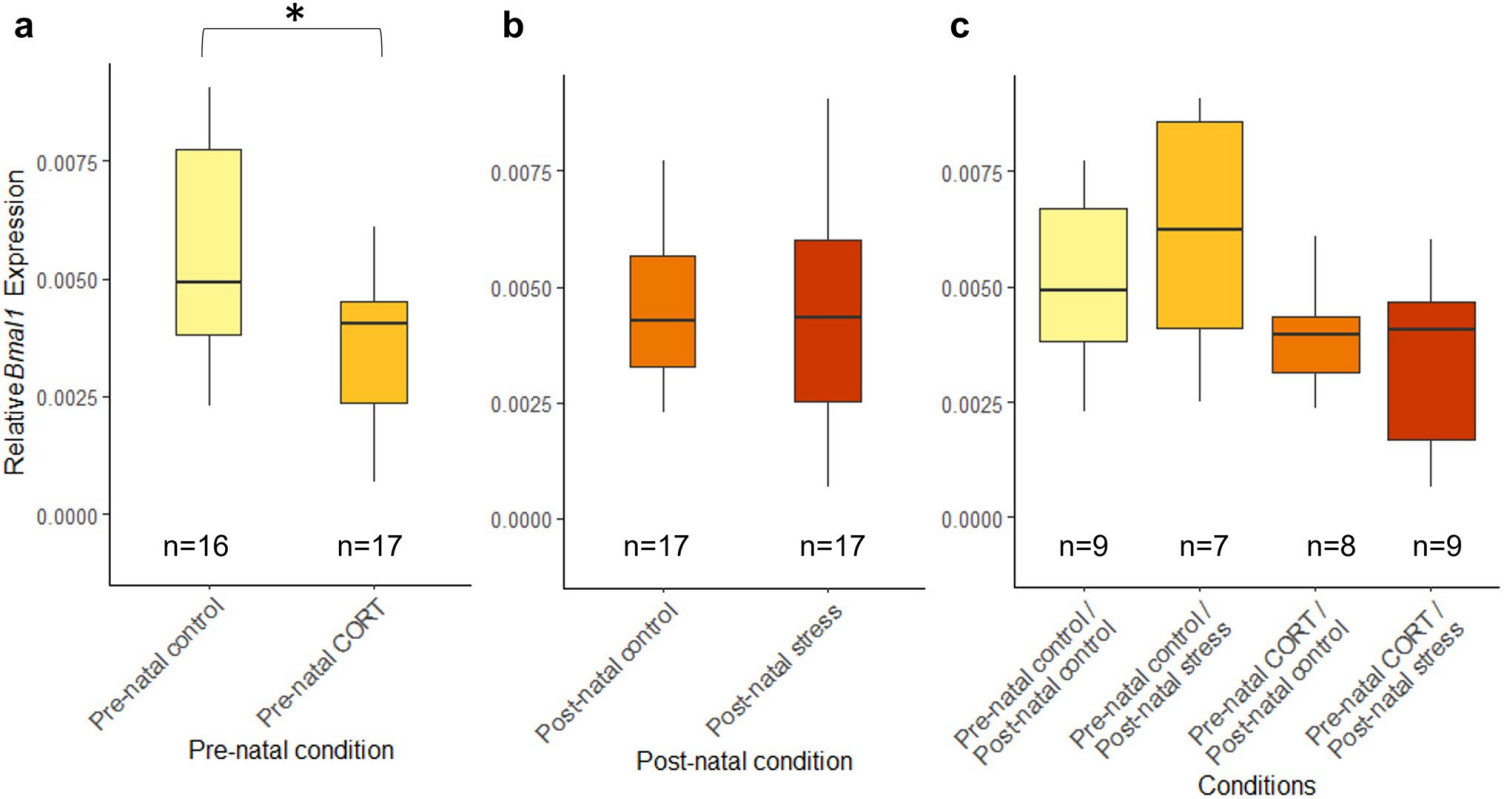
Effects of maternal developmental history on *Bmal1* gene deposition *at* HH15 using whole embryos. Normalized, untransformed Delta CT values are shown. **a** Pre-natal CORT exposure only. **b** Post-natal stress only. **c** Interactions between pre-natal CORT exposure and post-natal stress. Boxplots depict median relative expression levels and the 25th and 75th percentiles. Whiskers are 1.5X the interquartile range, data points outside this range are marked as outliers (circles). ***Indicate significant difference (*P* < 0.001), **Indicate significant differences (*P* < 0.01) and *Indicates significant differences (*P* < 0.05)

**Fig. 10:**
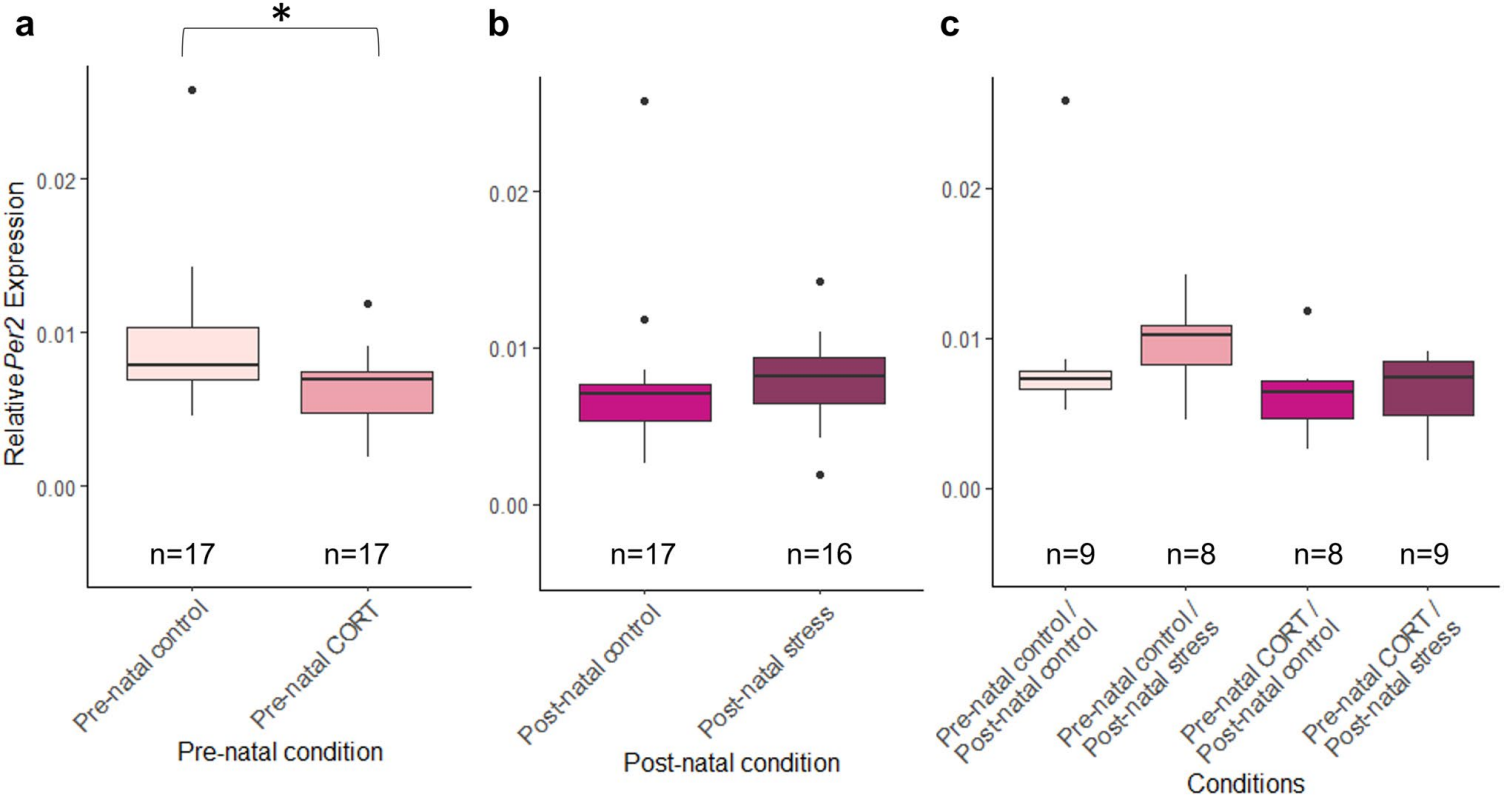
Effects of maternal developmental history on *Per2* gene deposition *at* HH15 using whole embryos. Normalized, untransformed Delta CT values are shown. **a** Pre-natal CORT exposure only. **b** Post-natal stress only. **c** Interactions between pre-natal CORT exposure and post-natal stress. Boxplots depict median relative expression levels and the 25th and 75th percentiles. Whiskers are 1.5X the interquartile range, data points outside this range are marked as outliers (circles). ***Indicate significant difference (*P* < 0.001), **Indicate significant differences (*P* < 0.01) and *Indicates significant differences (*P* < 0.05)

By HH15, levels of *Bmal1* within the dorsal region of the embryo were not found to be significantly affected by interactions between maternal pre- and post-natal stress (*F*_(1,30)_ = 1.89, *P* = 0.18, Fig. 9). *Bmal1* levels were, however, found to be significantly decreased if the mother had been exposed to pre-natal CORT (*F*_(1,30)_ = 7.13, *P* = 0.012). No significant effect of post-natal stress alone was identified (*F*_(1,30)_ = 0.2, *P* = 0.66). *Per2* featured the same patterns of expression; no significant interaction between maternal pre-natal and post-natal stress identified (*F*_(1,29)_ = 0.13, *P* = 0.72, Fig. 10). A significant decrease was present following pre-natal maternal CORT exposure (*F*_(1,29)_ = 5.86, *P* = 0.022), and no significant differences in *Per2* expression were identified for post-natal stress (*F*_(1,29)_ = 0.37, *P* = 0.29).

## Discussion

**Fig. 11:**
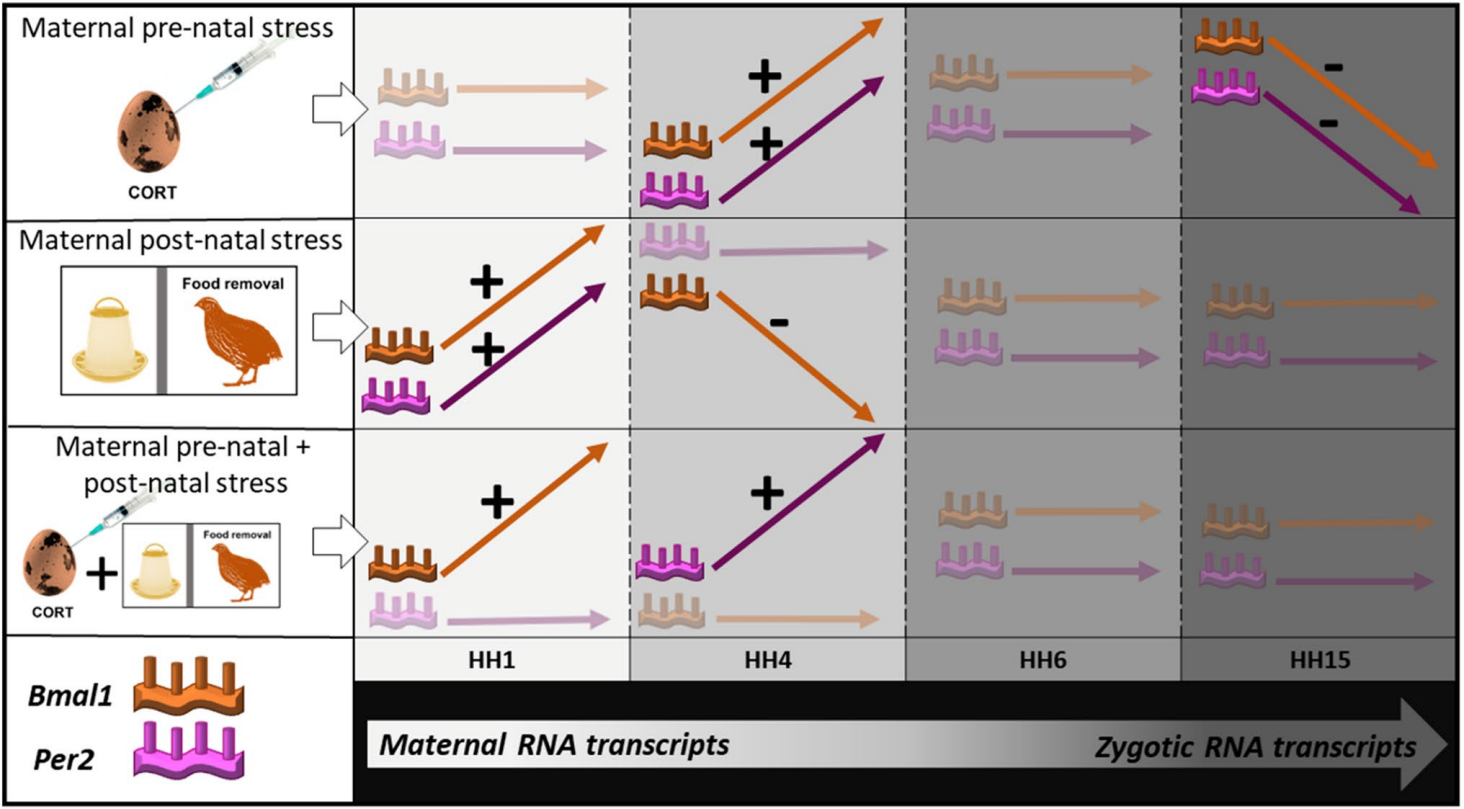
Graphical depiction of results. Mothers exposed to pre-natal stress only were not found to deposit different levels of *Bmal1* and *Per2* transcripts *in ovo* (HH1)*.* Embryos of mothers exposed to pre-natal stress only were found to have significantly increased levels of both *Bmal1* and *Per2* compared to controls at HH4. No differences were identified at HH6. At HH15 embryos featured significantly decreased *Bmal1* and *Per2. M*others who had experienced post-natal stress in early life were found to deposit *in ovo* (HH1) higher levels of *Bmal1* and *Per2* transcripts than controls. At HH4 *Bmal1* levels were significantly decreased when compared to controls. No further differences were identified at HH6 and HH15. Mothers who experienced both pre-natal and post-natal stress were found to initially deposit larger quantities of *Bmal1* when compared to all other conditions. At HH4, increased levels of *Per2* were identified when compared to mothers who had experienced post-natal stress only. No further differences were found at HH6 and HH15

We present the first evidence that environmental conditions experienced by the mother can drive later RNA deposition and ontogenesis of circadian clock genes, and in turn early embryonic transcription of circadian clock genes (Fig. 11). Pre-natal stress appears to feature longer term modifications to the embryonic genome, whilst post-natal stress elicits a change in maternal gene deposition which appears more transient in nature. Such pre-natal communication of environmental information between parents and offspring offers a fitness advantage, by ‘preparing’ the offspring for a specific post-natal environment (Dzialowski and Sotherland 2004; Horner and Wolfner 2008; Hargitai et al. 2009; Abrams and Mullins 2009; Sharda et al. 2021). This may then give rise to adaptive potential by phenotypic alterations which are appropriate for the maternally communicated environment (Mousseau and Fox 1998; Räsänen and Kruuk 2007; Shama et al. 2014; English et al. 2015; Kuijper and Hoyle 2015; Van Dooren et al. 2016; Lubzens et al. 2017). This is further supported by both pre-natal and post-natal stress elicit cumulative increases in initial *Bmal1* expression, and *Per2* expression during initial embryonic transcription (HH1 and HH4). Communicating environments through non-genomic means is a relatively new avenue of research; therefore, the full extent of non-genomic maternal communication in shaping adaptive potential is a largely unknown (Kuijper and Hoyle 2015; Moore et al. 2019). The downstream effects of the altered circadian expression identified in this study remain unknown and further studies investigating post-natal circadian hormone activity and behaviour should be conducted to confirm if these mRNA findings translate to a meaningful biological response.

### Post-natal maternal stress elicits changes in Circadian clock gene RNA deposition

Post-natal maternal stress was found to elicit transient changes in circadian RNA levels, with all differences disappearing by HH6. Goncalves et al., have previously confirmed circadian RNA at these early developmental timepoints are maternally deposited (Gonçalves et al. 2012). The altered levels of both *Bmal1* and *Per2* we identified provides strong evidence that post-natal maternal stress altered levels of maternally deposited circadian clock gene RNA *in ovo*. For initial maternal transcripts, at HH1, both *Bmal1* and *Per2* were found to be significantly increased in embryos from mothers who had experienced post-natal stress exposure, but not from those who had experiences pre-natal stress. Due to the immature state of the circadian system at this point in development the autoinhibitory expression of both *Bmal1* and *Per2* is not yet fully functional (Okabayashi et al. 2003). It is, therefore, not surprising that both the positive and negative regulator are increased in expression.

The increased expression of both *Bmal1* and Per2 from mothers experiencing post-natal stress only, suggests that stress features different effects on maternal transmission depending on the developmental time (pre- or post-natal) it was experienced. A significant interaction between pre-natal and post-natal stress was present for *Bmal1* expression. *Bmal1* was significantly increased in pre-natal CORT/post-natal stress compared to all other conditions.

At HH4, *Bma1* expression was again altered following post-natal maternal stress. A significant decrease in *Bmal1* expression was identified. *Per2* expression was unaffected. Pre-natal and post-natal maternal stress significantly interacted at HH4; both pre-natal and post-natal stress resulted in a higher expression of *Per2* compared to post-natal stress alone. It is thought the majority of embryonic circadian clock genes become active at HH6 (Gonçalves et al. 2012). At this point, maternal developmental history was not found to alter *Bmal1* or *Per2* expression.

### Pre-natal maternal stress causes changes to embryonic circadian transcript levels

Pre-natal maternal stress appears to have a delayed effect on circadian clock gene expression. This is likely due to changes in the embryonic genome (which gradually activates) rather than alteration to initial maternal transcript deposits. Pre-natal maternal stress effects became apparent at HH4. Both *Bmal1* and *Per2* were found to be significantly increased following maternal exposure to pre-natal stress. Maternal to zygotic genome activation occurs in gradual steps; therefore, it is not unlikely this may indicate changes in the embryonic genome. By HH15 the embryo is presumed to be transcribing only the embryonic genome. Pre-natal stress caused a significant decrease in both *Bmal1* and *Per2* expression. Again given the immature state of the autoinhibitory feedback loop of these genes it is not surprising the same effects were seen in both.

### Pre-natal and post-natal stress elicit cumulative increases in circadian clock gene RNA

Exposure to both pre- and post-natal stress appears to elicit a cumulative increase in maternal *Bmal1* deposition. *Per2* did not display such increases, indicating the maternal *Bmal1* deposition is most susceptible to environmental stress. The increased levels of *Bmal1* may then alter the phase of the embryonic circadian clock. Synchronization of embryonic clocks have been found to be altered by different levels of maternally deposited *Per3* in zebrafish (Delaunay et al. 2000). The cumulative effect of stress may be a method of communicating reliably stressful maternal environments to offspring. Such cumulative stress effects were also seen in *Per2* at HH4; maternal pre-natal and post-natal stress elicited significantly higher *Per2* levels compared to post-natal stress alone.

Taken together the results suggest that the developmental period a stress is received alters the quantities of maternal circadian RNA deposited in eggs. Post-natal stress elicits a significant increase in circadian gene deposition. These differences disappear as the embryo begins to transcribe the embryonic genome. Pre-natal stress alone, however, features little impact until the end of maternal RNA transcription at HH4, where a significant increase is present in both genes. Pre-natal maternal stress appears to elicit long term effects on embryonic circadian gene transcription; significantly reducing the amount synthesised at HH15. This may indicate maternally experienced embryonic stress causes a more permanent change in future maternal communication than post-natal stress. This may be due to epigenetic modifications to the embryonic genome (Aiken and Ozanne 2014) which are not communicated through varying transcript deposition levels, and do not appear until activation of the embryonic genome. This would contrast to post-natal developmental stress which is communicated by varying levels of maternal RNA.

Quail who received both pre-natal and post-natal stress deposited significantly higher amounts of transcripts into the embryo. This suggests that the type of stress received influences maternal deposition of mRNA; consistent stress (from pre-natal CORT/post-natal stress) is better communicated to the embryo, compared to inconsistent stress (from pre-natal control/post-natal stress or pre-natal control/post-natal stress).

Recent studies have found *in ovo* development to be more flexible than previously thought. Signals during incubation, such as acoustics, clutch size and incubation patterns can alter post-natal phenotypes (Gorman and Nager 2004; Reid et al. 2008; Noguera 2019; Mariette 2020). Such external factors likely effect the cumulative stress effects seen in this experiment and may potentially reinforce altered levels of circadian clock genes, allowing effects seen to persist to embryonic transcription.

For the most part, however, maternal signals are communicated before oviposition in the form of RNA, proteins and hormones (Ahi et al. 2018; Costantini et al. 2014; Monaghan 2007; Valcu et al. 2019). It is thought for optimal survival of offspring, the ‘one off’ signals put into the egg need to reliably ‘predict’ the future post-natal environment. Maternal signals can rapidly alter offspring phenotypes (Räsänen and Kruuk. 2007). This is thought to be problematic when in an unpredictable environment. A caveat of this rapid alteration is offspring may be altered to optimize fitness in one extreme post-natal environment and may hatch into the opposite, reducing fitness (Monaghan 2007). Unlike placental development there is no continuous biochemical communication from the mother (Groothuis et al. 2019, 2020). Oviparous maternal signals need to reliably predict the future post-natal environment without direct maternal feedback.

To date there is little research on the transgenerational effects of stress and the circadian system. A recent study in mice showed that offspring from mothers exposed to chronic stress during pregnancy had advanced circadian phase behaviour, increased *PER1* Suprachiasmatic nuclei (SCN) expression and finally significant variation in activity onset (Yun et al. 2020). Other findings in mammals confirm that stress exposure during pregnancy alters offspring circadian behaviour. Alterations included fragmented sleep and phase advance in both activity and corticosterone rhythm (Koehl et al. 1997; Dugovic et al. 1999; Kiryanova et al. 2017; Morley-Fletcher et al. 2019). Currently there is no research into maternal developmental history and the effects on offspring circadian gene expression in oviparous organisms. Our results suggest that maternal circadian gene transfer is altered if the maternal life history consists of both a pre-natal exposure to CORT, and a post-natal stressor.

If the environment of an embryo predisposed to altered circadian clock genes is poor this may signal for altered regulation. Altered circadian clock gene expression is generally regarded as detrimental to long-term health (Rijo-Ferreira and Takahashi 2019). Altered levels of *PER2* are associated with altered circadian activity, such as disrupted sleep rhythms and phase delays (Balsalobre et al. 2000; Chen et al. 2009; Cheon et al. 2013). This may, however, be beneficial to survival in a poor environment. A disrupted rhythm may increase fitness when there are no predictable sources of necessities, for example, food and temperature. As an example, if the organism follows predictable diurnal rhythms, but food is unpredictable, this may decrease chances of obtaining food (by sleeping when sporadic food becomes present). In addition, if the poor environment correlates with increased predators, it may be beneficial to rest for short periods interspersed with moving to a new location, compared to constant sleep for 8 h. This gives a short-term advantage to survival and is, therefore, not selected against as the animal will still reproduce. The disrupted rhythms may act as a trade-off between reaching sexual maturity and reproducing with a shortened lifespan. This ‘grow now pay later’ trade-off is known to occur from other maternal signals (Metcalfe and Monaghan 2001; Monaghan 2007).

### Supplementary Information

Below is the link to the electronic supplementary material.

Supplementary file1 (DOCX 14 kb)

## Data Availability

Research data and R analysis code is available at DOI: 10.17630/792c7a01-54d3-48dd-8588-b29bbee39608.
