# Supplementary material for: Maternal developmental history alters transfer of circadian clock genes to offspring in Japanese quail (Coturnix japonica)
Source: J Comp Physiol A Neuroethol Sens Neural Behav Physiol. 2023 Aug 17;210(3):399–413. doi: 10.1007/s00359-023-01666-2 (PMC11106187; doi:10.1007/s00359-023-01666-2)
Supplement: Supplementary file 1 — Supplementary file1 (DOCX 14 kb) [file 359_2023_1666_MOESM1_ESM.docx]

Supplemental material

| Table 1. Non-significant *post hoc* results for pre-natal/ post-natal interactions. | | | |
| --- | --- | --- | --- |
| Gene | Stage | Interactions (pre-natal:post-natal) | p value |
| *Bmal1* | HH1 | CORT:control-control:control | 0.38 |
| *BmalL1* | HH1 | control:stress-control:control | 0.97 |
| *Bmal1* | HH1 | control:stress-CORT:control | 0.59 |
| *Per2* | HH4 | CORT:control-control:control | 0.96 |
| *Per2* | HH4 | control:stress-control:control | 0.18 |
| *Per2* | HH4 | Control:stress-CORT:control | 0.067 |
| *Per2* | HH4 | CORT:stress-control:control | 0.29 |
| *Per2* | HH4 | CORT:stress-CORT:control | 0.58 |
